# Characterization of a Genogroup I Sapovirus Isolated from Chimpanzees in the Republic of Congo

**DOI:** 10.1128/genomeA.00680-14

**Published:** 2014-07-17

**Authors:** Illich M. Mombo, Nicolas Berthet, Christiane Bouchier, Joseph N. Fair, Bradley S. Schneider, François Renaud, Eric M. Leroy, Virginie Rougeron

**Affiliations:** a International Center for Medical Research of Franceville, Franceville, Gabon; b Laboratoire MIVEGEC UMR 224–5290 CNRS-IRD-UM1-UM2, IRD Montpellier, Montpellier, France; c Centre National de la Recherche Scientifique, UMR3569, Paris, France; d Metabiota, San Francisco, California, USA; e CHRU de Montpellier, Montpellier, France

## Abstract

Sapoviruses, which are members of the *Caliciviridae* family, are small nonenveloped viruses known to infect a large spectrum of mammalian hosts. We report here the first complete genome sequences of two genogroup I sapoviruses isolated from fecal samples from chimpanzees living in the Tchimpounga sanctuary, Republic of Congo.

## GENOME ANNOUNCEMENT

Sapoviruses (SaVs), which are members of the *Caliciviridae* family, are small nonenveloped viruses known to infect a large spectrum of mammalian hosts, such as humans, minks, sea lions, swine, dogs, and bats. SaVs are responsible of gastroenteritis in humans (1). Their genome, a nonsegmented positive-sense single-strand RNA, is 7.5 to 8.5 kb in length. It contains two or three open reading frames (ORFs) (2). Based on the complete capsid gene, SaVs are classified into 14 genogroups (GI to GXIV) (3). Currently, full-length genomes are available for GI to GVII and GXIV SaVs, all obtained from human fecal samples isolated from several parts of the world, except Africa. Here, we report the complete genome of SaVs (Cpz-IJC04 and Cpz-IJC09) identified in two chimpanzee (*Pan troglodytes troglodytes*) fecal samples from primates living in the Tchimpounga sanctuary (Republic of Congo) that displayed no gastrointestinal symptoms.

Since this is the first detection of sapoviruses in nonhuman primates living in close contact with humans, we investigated their full genomes. Unbiased deep sequencing employing an Illumina HiSeq platform was conducted as follows: extracted RNA was treated with Turbo DNase (Life Technologies), reverse transcribed (RT) using random hexamers (Life Technologies), and amplified using Phi29 enzyme (4). Contigs were assembled with ABySS software (5) and the CAP3 program (6) to construct the viral genomes. The ORFs were identified using the ORF Finder (http://www.ncbi.nlm.nih.gov/projects/gorf/gorf.html).

Despite repeated analysis, the 5′ untranslated region (UTR) (10 nucleotides [nt]) and 3′ UTR [28 nt] were not obtained. The assembled genomes consist of 7,320 nt and contain three presumptive ORFs: nucleotide positions 1 to 6837 (ORF1), 6840 to 7319 (partial ORF2), and 5168 to 5653 (ORF3). An examination of the 2,280 amino acids of ORF1 revealed that these genomes contain motifs that are characteristic of caliciviruses: a 2C-like NTPase at residue 480 (GAPGIGKT), VPg at residues 951 (KGKTK) and 962 (DEYDE), a protease at residue 1167 (GDCG), RNA polymerase at residues 1503 (GLPSG) and 1551 (YGDD), and a VP1 at residue 1740 (PPG). Based on the deduced amino acid sequences of the capsid gene (4,530 to 6,843 nt), Cpz-IJC04 and Cpz-IJC09 are 100% identical, cluster within the human GI clade, and are closest (99.7% identity) to the GI human enteric calicivirus Plymouth isolate (GenBank accession no. X86559) (7).

The clustering of these two genomes within the human GI clade and the knowledge that these chimpanzees were living in close contact to humans (in the sanctuary) suggest recent cross-species transmission of these viruses from humans to chimpanzees. However, further analysis is required to confirm this hypothesis. It is important to investigate the prevalence of these viruses in wild chimpanzee populations in order to obtain a better understanding of their evolution and adaptation in these animals. The acquisition of these new SaV genomes will facilitate the design of new specific primer panels for reverse transcription-PCR (RT-PCR) assays, allowing a better understanding of the epidemiology and potential pathogenicity of these SaVs.

### Nucleotide sequence accession numbers.

The sequenced genomes of Cpz-IJC04 and Cpz-IJC09 have been submitted to GenBank under accession no. KJ858686 and KJ858687, respectively.
